# Hepatitis D in the Middle East: A Review of Disease Burden, Health-System Barriers, and Strategic Directions for Management and Elimination

**DOI:** 10.7759/cureus.108277

**Published:** 2026-05-04

**Authors:** Maryam Alkhatry, Abdul J Alwan, Adel Qutub, Adnan Alzanbagi, Fozeya Alzaabi, Huda Al Quraishi, Maen Al Mattooq, Mazen Taha, Muneera Y Altarrah, Nawal Alnahdi, Saeed Husain Al Marzooqi, Yehia Nabil, Faisal Aba-Alkhail

**Affiliations:** 1 Gastrointestinal Surgery and Endoscopy Department, Ibrahim Bin Hamad Obaidullah Hospital, UAE, ARE; 2 Liver Transplant and Hepatology Department, International Medical Research Centre, Ministry of National Guard Health, Riyadh, SAU; 3 Gastroenterology and Hepatology Department, King Fahad Medical City, Riyadh, SAU; 4 Gastroenterology and Hepatology Department, King Abdullah Medical City, Makkah, SAU; 5 Gastroenterology and Hepatology Department, Zayed Military Hospital, Abu Dhabi, ARE; 6 Gastroenterology and Hepatology Department, Rashid Hospital, Dubai, ARE; 7 Gastroenterology and Hepatology Department, Mohammed Bin Rashid University of Medicine and Health Sciences, Dubai, ARE; 8 Gastroenterology and Transplant Hepatology Department, Jaber Al Ahmad Hospital, Kuwait, KWT; 9 Gastroenterology and Hepatology Department, Tawam Hospital, Abu Dhabi, ARE; 10 Gastroenterology and Transplant Hepatology Department, Al-Amiri Hospital, Kuwait, KWT; 11 Gastroenterology, Hepatology and Endoscopy Department, Rashid Hospital, Dubai, ARE; 12 Gastroenterology and Hepatology Department, Sheikh Shakhbout Medical City, Abu Dhabi, ARE; 13 Medical Affairs Department, Gilead Sciences Inc., Dubai, ARE; 14 Medicine Department, King Faisal Specialist Hospital and Research Centre, Riyadh, SAU

**Keywords:** bulevirtide, hepatitis b virus, hepatitis delta virus, lonafarnib, rna testing

## Abstract

Hepatitis delta virus (HDV) is the most aggressive form of viral hepatitis and worsens outcomes in people with chronic hepatitis B (HBV). It speeds up fibrosis, causes early cirrhosis, hepatic decompensation, and increases hepatocellular carcinoma risk. In the Middle East, presumed high HBV prevalence, uneven surveillance, and significant diagnostic and treatment barriers lead to under-recognition of HDV and uncertainty about its true prevalence. This review summarizes current regional evidence on HDV distribution, diagnostic gaps, emerging treatments, and health policy shortcomings. The results show wide variation in prevalence, with some populations exhibiting endemic levels and others lacking reliable screening data due to limited testing and limited availability of HDV RNA. Diagnostic issues are exacerbated by high costs, limited laboratory resources, gaps in provider awareness, and delayed patient presentation. While new therapies like bulevirtide and lonafarnib show promising virologic and biochemical results, access remains limited due to regulatory delays, costs, and fragmented care. Policy analysis finds HDV is rarely prioritized in national hepatitis plans, leading to weak surveillance, poor registry systems, and little focus on high-risk groups. Hence, improving routine screening for HBsAg-positive individuals, expanding access to affordable RNA testing, increasing access to approved and emerging therapies, and establishing integrated HBV-HDV management protocols are vital for reducing the disease burden. Including HDV in national elimination efforts, boosting surveillance, and enhancing workforce capacity are crucial steps to meet the WHO 2030 hepatitis goals and curb HDV’s growing public health impact in the Middle East.

## Introduction and background

Hepatitis delta virus (HDV) is one of the most severe forms of viral hepatitis, impacting around 12 million people globally [1]. It can be transmitted through infected blood, body fluids, sexual contact, or, in rare cases, from mother to child during birth [2]. HDV is a defective, single-stranded circular RNA virus belonging to the genus Deltavirus. As a satellite virus, HDV relies heavily on the Hepatitis B virus (HBV) for both replication and assembly, so the endemic status of HBV is a critical determinant of HDV occurrence. HDV requires HBV surface antigen (HBsAg) to assemble infectious particles to enter hepatocytes [3]. This unique dependency explains why HDV infection is found only in individuals with HBV, either as co-infection or superinfection. Co-infection often results in severe acute hepatitis, while superinfection in chronic HBV accelerates the progression to advanced liver disease (ALD). Emerging evidence indicates that HDV can disrupt host immune mechanisms and worsen underlying liver inflammation, highlighting its clinical significance. HDV infection has been linked to the fastest progression to cirrhosis among viral hepatitis, with nearly 70-80% of patients developing cirrhosis over a period of 5-10 years [4-6]. Compared to HBV alone, co-infection markedly increases the risk of hepatic cirrhosis and hepatocellular carcinoma (HCC) [6,7].

Global prevalence estimates of anti-HDV positivity vary considerably. According to a systematic review and meta-analysis involving 282 studies from 95 countries and 120,293 individuals, HDV prevalence was 4.5% among HBsAg-positive individuals and 16.4% among individuals who visit hepatology clinics, indicating significant heterogeneity in disease distribution. Moreover, the study also reported the high prevalence in Mongolia, the Republic of Moldova, and Western and Middle Africa, particularly among high-risk groups. Further, the study analyzed HDV RNA in 5,073 anti-HDV-positive patients and found that the pooled detection rate was 58.5% [1]. In North Africa, the general population has a prevalence of 5.01%, which increases to 20.7% among patients with liver disease [8]. In South America, the burden of this disease is particularly high, with pooled prevalence reaching 22.37% among HBV carriers [9]. However, these estimates underscore substantial geographical heterogeneity and highlight important gaps in region-specific epidemiological data, particularly in underrepresented regions. Data on HDV prevalence in some Middle Eastern countries remain limited.

Despite the substantial impact of the HDV, standardized monitoring guidelines for diagnosed cases remain limited. Recent advances in serological assays and non-invasive screening strategies are reshaping HDV management and improving diagnostic capacity [10]. Nevertheless, the lack of uniform screening strategies, limited awareness, and variability in diagnostic practices continue to impede accurate estimation of disease burden and timely clinical management. At the healthcare system level, limited clinical awareness, the persistent perception of HDV as a rare disease confined to high-risk groups, and the absence of reliable diagnostic assays have hindered an accurate appraisal of HDV’s global burden [11]. At the policy level, testing recommendations vary across regions, leading to inconsistent clinical practice. While the European Association for the Study of the Liver (EASL) and the Asian Pacific Association for the Study of the Liver (APASL) advocate screening all HBsAg-positive individuals for anti-HDV antibodies, the American Association for the Study of Liver Diseases (AASLD) restricts testing to high-risk populations. Furthermore, at the diagnostic level, practices remain inconsistent, as many antibody-positive cases are not followed by HDV RNA confirmation, and RNA quantification itself lacks standardization, leading to variability across assays [10-12].

In the Middle East, data on HDV prevalence are inconsistent, and the regional distribution as well as the overall burden of the disease are not well-documented [5]. This lack of consolidated, region-specific evidence limits informed policy-making and targeted intervention strategies, highlighting a critical research gap in the synthesis of epidemiological, diagnostic, and management data across the region. Enhancing the evidence base is vital for reaching the WHO’s 2030 elimination targets, which aim to eliminate 9.5 million new infections and save 2.8 million lives [13]. However, the applicability and progress toward these targets in the Middle Eastern setting remain insufficiently characterized due to fragmented data and variable healthcare infrastructure. Accordingly, this narrative review aims to provide a critical and region-specific synthesis of the HDV landscape in the Middle East, delineating the disease burden, understanding the gaps in diagnosis, treatment, and policy, and underscoring the urgent necessity for coordinated strategies to strengthen HDV control and advance regional progress toward global hepatitis elimination goals.

## Review

Methodology

This narrative review was conducted to synthesize evidence on HDV epidemiology, diagnosis, treatment, and policy in the Middle East, using a structured approach to ensure transparency and reproducibility. A comprehensive search was conducted in PubMed and Google Scholar for publications from January 2015 to November 2025, using combinations of key terms, such as “Hepatitis D virus prevalence” and “HDV epidemiology Middle East.” Diagnostic terms comprised “HDV diagnosis” and “HDV screening guidelines. Treatment-related searches used “HDV treatment outcomes Middle East” and “HDV investigational therapies.” Policy and health system terms included “HDV surveillance Middle East,” “WHO EMRO hepatitis guidelines,” and “national hepatitis programs Middle East.” Country-specific searches were conducted for Iran, Iraq, Saudi Arabia, Turkey, Egypt, Yemen, United Arab Emirates, Qatar, Oman, Bahrain, Jordan, Lebanon, Syria, and Kuwait to capture heterogeneity in prevalence and practice. Studies were included if they reported HDV epidemiology, diagnostic practices, treatment outcomes, or policy frameworks in the Middle East; exclusions comprised non-human studies and outdated publications.

Epidemiology

Global Epidemiology

HDV, although dependent on HBV for replication, represents a severe acute and chronic viral hepatitis in humans, and its prevalence remains globally variable. It is estimated to affect 0.16% of the worldwide population (approximately 12 million), with HDV contributing to 18% and 20% of cirrhosis and HCC cases, respectively [1,5,14]. The risk of HDV infection is concentrated among persons who inject drugs (PWID), hemodialysis recipients, men having sex with men, refugees/migrants, and individuals with HIV/Hepatitis C virus (HCV) coinfection. Among HBsAg-positive populations, the pooled prevalence of HDV antibody positivity is 14.57%, ranging from 10.58% in mixed populations without intravenous drug use (IVDU) or high-risk sexual behavior (HRSB) to 37.57% in IVDU groups and 17.01% in HRSB groups [1,15-17]. This wide variation across risk groups highlights the strong influence of behavioral and exposure-related factors on HDV transmission dynamics. HDV is more prevalent in Eastern Europe, Central Asia, Central and West Sub-Saharan Africa, as well as in Tropical and Central Latin America. Asia exhibits antibody prevalence rates ranging from 44.41% to 56.55%, while Africa ranges from 22.3% to 33.87% [6]. Such geographic heterogeneity likely reflects differences in screening practices, HBV endemicity, and public health infrastructure across regions. According to a US-based study conducted on 144,975 patients with HBV, 6719 (4.6%) patients were diagnosed with HDV co-infection [18].

Regional Epidemiology

Studies from Iran reported a variable prevalence of HDV, ranging from 0.0% to 0.53% in the general population and from 0.0% to 21.8% among HBV-positive patients [5]. This marked intra-country variability suggests differences in study populations, diagnostic approaches, and regional risk profiles. Furthermore, in a single-center study in Saudi Arabia involving 169 HBsAg-positive patients, 7.7% (n=13) were found to be HDV antibody-positive [19]. Another study from Lebanon, conducted on 258 HBsAg-positive patients collected from 10 medical centres, reported the prevalence of the delta antibody among HBsAg-positive patients to be around 1% [20].

In Egypt, a prospective study among 186 HBsAg-positive patients reported seropositivity for anti-HDV in 43% of patients [21]. Moreover, in 2015, a study conducted in Iraq found that 6.6% of individuals with HBV (n=45) tested positive for anti-HDV antibodies [22]. In contrast to this, a study conducted in Yemen screened 501 individuals for HDV, HBV, and HCV, but none of them were found to be positive for HDV antibodies [23]. Collectively, these findings demonstrate substantial inter-country heterogeneity, ranging from negligible to very high prevalence, which may be attributed to differences in sample size, population selection, and diagnostic sensitivity. However, several countries like the UAE, Qatar, Bahrain, and Syria have not identified prevalence data in the general population. WHO has reported limited data on the diagnosis and treatment of HDV in the Eastern Mediterranean Region. This overall variability and data paucity underscore the challenge of accurately estimating regional disease burden. This highlights broader issues such as underdiagnosis caused by limited access to diagnostic tests, limited awareness of HDV, and non-standardized HDV RNA testing [5,10,16,24-26].

Clinical Consequences

Chronic HDV infection accelerates liver disease progression compared with HBV alone, leading to cirrhosis onset. Nearly 30% to 70% of chronic HDV patients are detected with cirrhosis at diagnosis, and over 50% mortality is reported within 10 years of liver disease diagnosis [8,11,25]. This aggressive clinical course further emphasizes the importance of early detection and consistent epidemiological surveillance. In patients with ALD, patient monitoring is needed for portal hypertension, hepatic decompensation, and hepatocellular carcinoma development. Consequently, recognition of these complications warrants specific treatments and timely intervention, including liver transplant [11].

Diagnostic landscape and regional gaps

Screening for HDV is implemented by detecting anti-HDV antibodies, with IgG remaining positive after exposure, even post-clearance [27]. It necessitates a sequential approach, starting with anti-HDV antibody screening, followed by HDV RNA testing to confirm active infection [11,28]. International guidance has converged towards routine HDV screening in all HBsAg-positive individuals. The EASL and APASL recommend universal screening. In contrast, the AASLD emphasizes a risk-based strategy, stating that HDV testing should be considered for HBsAg-positive patients with elevated transaminase levels and low or undetectable HBV DNA [29,30]. However, assays for HDV antigen and IgM anti-HDV are not widely available, while HDV RNA assays lack standardization. Low clinician awareness, limited availability of HDV RNA and antibody testing, and absence of reimbursement widen the diagnostic gap, resulting in many patients being diagnosed only once ALD has developed [31]. Additionally, innovative approaches, such as point-of-care rapid diagnostic tests, are not yet available. Furthermore, assessing liver fibrosis in HDV remains challenging, as non-invasive tests are impractical [17].

HDV diagnosis in the Middle East remains limited [5]. Based on available data from Saudi Arabia, it is observed that systemic challenges, including fragmented healthcare infrastructure, inadequate surveillance, and a lack of effective public awareness campaigns, impact the transmission of viral hepatitis [32]. Furthermore, testing for HDV is limited, and RNA-based confirmation is often unavailable. These obstacles also include a lack of reflex testing procedures, standardization, and reliable non-invasive tests. Routine testing of all HBsAg-positive individuals, complemented by anti-HDV and HDV RNA testing, including the use of dry blood spots in representative population surveys, such as demographic health surveys, would strengthen epidemiological data in regions with limited information. Implementing laboratory reflex testing would expand the evidence base on HDV prevalence and facilitate earlier identification and referral to care for HDV RNA-positive individuals. New biomarkers capable of tracking disease progression are urgently needed [1,33,34].

Treatment and management landscape

Till recently, over the past 30 years, HDV infection management has largely remained unchanged, centered on interferon therapy [35]. HDV infection lacked an approved therapy, with pegIFN-α used only off-label. In 2020, the European Medical Association authorized bulevirtide for HDV-related chronic liver disease, marking the first officially approved treatment for this condition [36].

Bulevirtide (BLV), a sodium taurocholate co-transporting polypeptide entry inhibitor, has demonstrated notable virologic suppression. In the Phase III MYR301 trial, virological response (HDV RNA undetectable for ≥2 log10 IU/mL decline) occurred in 71% of patients on bulevirtide 2mg, vs 4% in controls. Among those with undetectable HDV RNA at ≥96 weeks, 90% maintained viral control after treatment cessation without relapse. However, further evaluation of long-term outcomes with single-agent therapy is still needed [37]. Importantly, much of the existing evidence, including clinical trial endpoints, relies on surrogate markers, such as HDV RNA decline, rather than hard clinical outcomes (e.g., progression to cirrhosis, hepatic decompensation, or mortality), thereby limiting definitive assessment of long-term clinical benefit. Additionally, Lonafarnib, a prenylation inhibitor, has also shown efficacy when combined with ritonavir. Preliminary results from the Phase 3 D-LIVR trial reported that one‑third of subjects achieved virologic or biochemical endpoints at 48 weeks [38]. However, similar to bulevirtide, these findings are based on relatively short follow-up durations, and robust long-term data on the durability of response and clinical outcomes remain limited. Nucleic acid polymers have also been found to possess antiviral activity in vitro and in early clinical studies by blocking virus entry [39]. Immunotherapy strategies, including prime‑boost vaccines and combination regimens, are currently under investigation to restore immune control [40, 41]. Overall, while these emerging therapies are promising, the current evidence base is still evolving, with a need for longer-term, real-world studies to establish sustained efficacy, safety, and impact on clinically meaningful endpoints. Detailed treatment options are presented in Table 1.

**Table 1:** Treatment options available for HDV Source: [42-47] ALT, alanine transferase; HBsAg, HBV surface antigen; HDV, hepatitis delta virus; PEG-IFN, pegylated interferon; RNA, ribonucleic acid

Author	Treatment	Administration	Mode of action	Efficacy	Phase
Heiner Wedemeyer et al., 2023 [42]	Bulevirtide	Subcutaneous	Inhibiting the viral entry within the hepatocytes	HDV RNA and ALT levels were reduced in patients with chronic hepatitis D.	Phase 3 - ongoing
Etzion et al 2023 [43]	Lonafarnib + Ritonavir	Oral	Prenylation inhibitor: blocks HDV virion assembly	10% of patients showed ≥2- log in HDV RNA decline, and 24% showed ALT normalization at 48 weeks	Phase 3 - ongoing
Etzion et al., 2023 [43]	Lonafarnib + Ritonavir ± Peg-IFN	Oral	Prenylation inhibitor: blocks HDV virion assembly	19 % of patients showed ≥2- log in HDV RNA decline and 34.4% showed ALT normalization at 48 weeks	Phase 3- ongoing
Shekhtman L et al., 2020 [44]	REP 2139-Ca (NAPs)	Intravenous	Blocks HBsAg release and HDV virion assembly	The efficacy of blocking HBsAg and HDV production were 98.2% and 99.7%	Modeling study
Asselah et al., 2024 [45]	Bulevirtide + Peg-IFN-α	Subcutaneous	Dual mechanism: entry inhibition + immune activation	Higher rates of undetectable HDV RNA 46% vs 12% with bulevirtide monotherapy	Phase 2
Al-Mansouri et al., 2025 [46]	PEG-IFN-lambda	Subcutaneous	Improving viral clearance and immune modulation	42% virological suppression by PEG-IFN-Lambda	Phase 2
Asselah T et al., 2025 [47]	Tobevibart plus Elebsiran	Subcutaneous	Boosting of HBsAg-targeting immunity and clearance of infected hepatic cells	Undetectable HDV RNA reported in 66% of patients	Phase 2 - ongoing

While bulevirtide marks a significant advance in HDV therapy, its widespread use is constrained by high costs and restrictive reimbursement policies, resulting in limited availability in a few countries. Peg‑IFNα is similarly constrained by modest efficacy and poor suitability, particularly in decompensated cirrhosis patients. Despite the effectiveness of newer therapies, the costs remain a major barrier to broad patient access [31,48].

Policy and health system gaps

**Figure 1:**
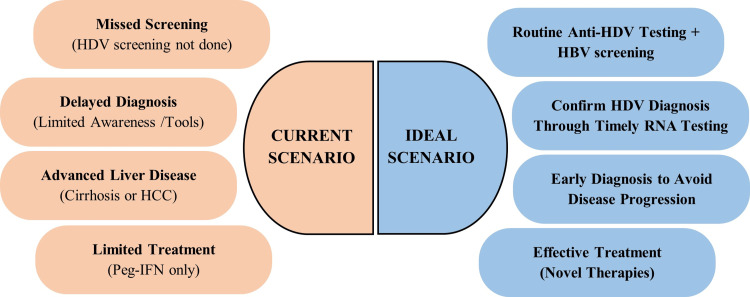
Current vs ideal patient journey scenario for HDV infection HDV, hepatitis delta virus; HBV, hepatitis B virus; RNA, ribonucleic acid; IFN, interferon; HCC, hepatocellular carcinoma

HDV is the most severe form of viral hepatitis, yet it is still not well-recognized or managed in many parts of the Middle East [5]. Awareness among patients and healthcare providers is still low, and HDV treatment has advanced far less than therapies for HBV and HCV [49]. Although advances in vaccination and treatment helped reduce prevalence, viral hepatitis remains a major challenge in Saudi Arabia, with diagnostic delays, healthcare gaps, and stigma slowing elimination efforts [32]. The few studies have indicated that regular checks are crucial to understand how often HBV patients also have HDV [32]. A comparison of the current and ideal HDV patient journey is depicted in Figure 1.

Middle Eastern and African (MEA) countries lack dedicated HDV elimination strategies, and hepatitis D is rarely prioritized within national hepatitis action plans. For example, the Lebanese Society of Gastroenterology has issued guidelines for HBV diagnosis without any specific preference for HDV [5]. Similarly, a 2016 review of hepatitis B and C in Syria noted that although testing and care for both infections were provided free of charge, no national strategy for HBV control or prevention guidelines existed, and HDV was not addressed [50]. Furthermore, in Oman, Iran, Iraq, Qatar, Bahrain, and Yemen, no national guidelines or policies for HBV or HDV were recognized [5]. Reliable tests are needed for the correct diagnosis of HDV infection and to assess treatment endpoints [33]. Diagnostic costs for HDV remain high, and patients frequently bear out-of-pocket expenses for both testing and treatment [5,51]. Approval processes for new therapeutics are slow, thereby restricting access to treatment [48].

HDV infection is often overlooked in the Middle East, underscoring the need for establishing patient registries and routine blood screening to improve recognition and control [33]. Focused efforts should be directed at medical trainees and primary care practices to enhance HDV screening uptake. Existing risk-based approaches fail to adequately identify infections, underscoring the need to shift toward universal HDV testing among individuals with chronic hepatitis B [52]. Reducing stigma requires public awareness, provider training, and community involvement to achieve better outcomes [32].

Actionable insights and strategic recommendations

**Figure 2:**
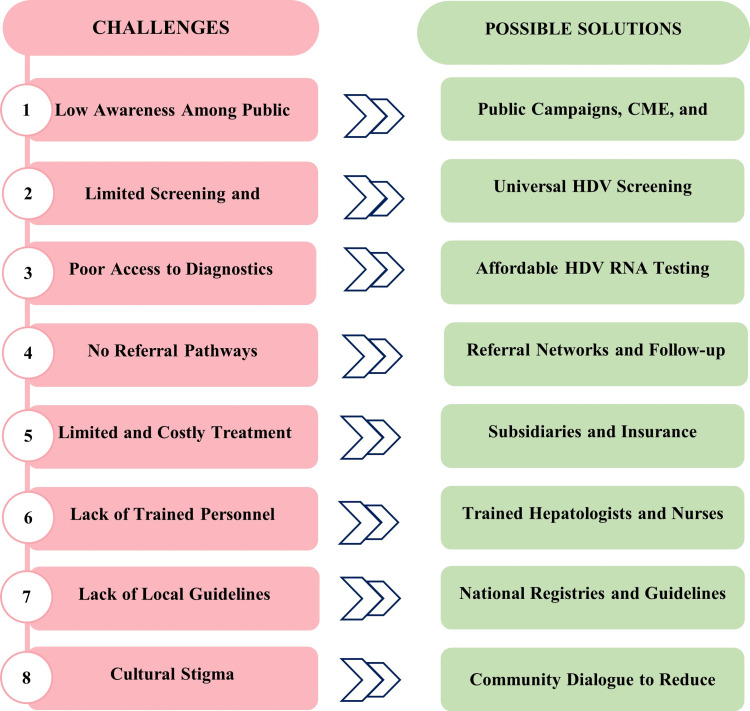
Challenges and corresponding solutions in HDV management in the Middle East CME, Continuing Medical Education; HCP, healthcare practitioner; HDV, hepatitis delta virus; RNA, ribonucleic acid

Addressing HDV in the Middle East requires a collaborative approach involving public health initiatives, enhanced healthcare infrastructure, revised policies, and active community participation. Figure 2 illustrates the key challenges and potential solutions for managing HDV in the region.

To maximize impact and feasibility, priority should be given to a few high-yield, implementable strategies, particularly in resource-variable settings. Universal screening for all HBsAg-positive patients is recommended, in accordance with the EASL, WHO, and APASL guidelines. EASL guidelines underscore the necessity of enhancing clinicians’ understanding of why testing for anti-HDV in HBsAg-positive carriers is crucial [2,53,54]. Reflex testing protocols can streamline the diagnosis by automatically confirming HDV RNA in antibody-positive cases [1,29]. A phased implementation of reflex testing, beginning in tertiary care or reference laboratories, could further enhance early detection while ensuring operational feasibility. It can help detect HDV earlier and enhance our understanding of its epidemiology [11]. Priority should be given to creating standardized HDV diagnostic assays that account for its genotypic variability and worldwide distribution [55]. Access to HDV RNA testing should be expanded, and reduced pricing can help patients combat financial barriers [5]. These diagnostic improvements should be aligned with scalable implementation strategies to avoid redundancy, with broader policy recommendations. Accelerated regulatory approval for new therapies is considered essential to improve treatment options [48]. Moreover, personalized counseling for patients with chronic hepatitis D can help slow or prevent liver disease progression [11]. WHO’s Global Health Sector Strategy (2022-2030) seeks to eliminate viral hepatitis by 2030 through a 90% reduction in new infections and a 65% reduction in mortality, achievable with expanded HBV vaccination, improved HBV/HCV prevention, testing, and treatment, and strengthened public awareness [17, 56]. Aligning regional strategies with these targets, while focusing on prioritized, high-impact interventions, may enhance both feasibility and measurable progress in the Middle Eastern context.

Proposed regional framework for HDV control

Raising awareness of HDV among clinicians and the public is crucial for early detection and stigma reduction [49]. Incorporating educational campaigns into national hepatitis initiatives can improve understanding of transmission risks and prevention strategies [2].

**Figure 3:**
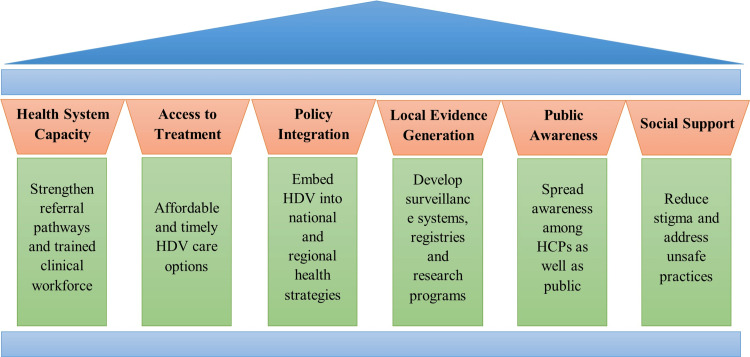
Core strategic pillars for addressing HDV across the Middle East HCPs, healthcare providers; HDV, hepatitis delta virus

Universal screening of all HBsAg-positive patients, followed by reflex HDV RNA confirmation, should be adopted region-wide. Furthermore, EASL, WHO, and APASL guidelines emphasize the importance of standardized diagnostic algorithms to ensure the prompt identification of active infection [11,13,26,29,53,54]. Expanding access to Peg-IFNα and expediting regulatory approval for newer treatments are essential [48]. Moreover, formulating and assessing the cost-effectiveness of HDV screening strategies depends on precise estimates of infection prevalence and the associated disease burden [29]. Establishing patient registries and routine blood screening is key to better identification and control [33]. The core strategic pillar for addressing HDV across the Middle East is shown in Figure 3.

Advancing towards the elimination of viral hepatitis requires decentralizing services and integrating them into existing healthcare platforms, such as HIV programs, to improve access for key and vulnerable populations facing structural barriers. Active participation of affected communities is essential to ensure that interventions are aligned with the epidemiological and social context. Global organizations emphasize advocacy, community engagement, and service delivery for priority groups. WHO has recommended strategies, such as engaging affected populations and civil society in advocacy and service delivery, strengthening national data systems and accountability through improved country-level information, expanding access to affordable, high-quality testing and diagnostics, and simplifying and decentralizing service delivery using a public health approach [57].

## Conclusions

HDV remains a severe yet substantially under-recognized cause of ALD in the Middle East and surrounding regions. This narrative review demonstrates that fragmented epidemiological data, limited routine screening of HBsAg-positive individuals, restricted access to HDV RNA testing, and delayed diagnosis, often at advanced stages of fibrosis, continue to drive poor clinical outcomes. These challenges are further compounded by gaps in national policies, inconsistent treatment availability, and persistent stigma, collectively hindering effective disease control and slowing progress toward global hepatitis elimination targets. The lack of insurance coverage for HDV treatment across many Middle Eastern countries further compounds the challenges of managing this severe infection.

In the future, the evolving therapeutic landscape, including agents such as bulevirtide and other emerging antiviral and immunomodulatory strategies, can offer a promising opportunity to transform HDV management. Realizing this potential will require the systematic integration of universal HDV screening into HBV programs, strengthened diagnostic and referral pathways, expanded and affordable access to novel therapies, addressing insurance gaps to ensure equitable access to care, and the generation of high-quality regional data through registries and population-based studies. With coordinated clinical, public health, and policy efforts, it is possible to shift from late detection and limited intervention toward earlier diagnosis, improved outcomes, and tangible progress toward achieving the WHO 2030 hepatitis elimination goals.
